# Retinal nerve fibre layer, and ganglion cell layer thicknesses in children with ADHD: associations with subtypes and symptom severity

**DOI:** 10.1186/s12888-026-08422-1

**Published:** 2026-08-07

**Authors:** Xiu Juan Zhang, Xiu Nian Chen, Yuzhou Zhang, Ka Wai Kam, Ian C. K. Wong, Wei Zhang, Alvin L. Young, Clement C. Tham, Chi Pui Pang, Li Jia Chen, Patrick Ip, Jason C. Yam

**Affiliations:** 1 Department of Ophthalmology and Visual Sciences, The Chinese University of Hong Kong, 4/F Department of Ophthalmology and Visual Sciences, Hong Kong Eye Hospital, 147K Argyle Street, Kowloon, Hong Kong SAR, China; 2 Department of Ophthalmology, Li Ka Shing Faculty of Medicine, The University of Hong Kong, Hong Kong SAR, China; 3 Department of Ophthalmology and Visual Sciences, Prince of Wales Hospital, Hong Kong SAR, China; 4 Centre for Safe Medication Practice and Research, Department of Pharmacology and Pharmacy, Li Ka Shing Faculty of Medicine, The University of Hong Kong, Hong Kong SAR, China; 5 Tianjin Eye Hospital, Tianjin, People’s Republic of China; 6 Hong Kong Eye Hospital, Hong Kong SAR, China; 7 Department of Ophthalmology, Hong Kong Children’s Hospital, Hong Kong SAR, China; 8 Hong Kong Hub of Paediatric Excellence, The Chinese University of Hong Kong, Hong Kong SAR, China; 9 Department of Paediatrics and Adolescent Medicine, Li Ka Shing, Faculty of Medicine, The University of Hong Kong, Hong Kong SAR, China

**Keywords:** ADHD subtypes, RNFL thickness, GCL thickness

## Abstract

**Background:**

To investigate retinal nerve fibre layer (RNFL) and ganglion cell layer (GCL) thickness in different attention-deficit/hyperactivity disorder (ADHD) subtypes and their association with ADHD symptom severity.

**Methods:**

This is a cross-sectional study included children aged 6–8 years in Hong Kong. ADHD diagnoses were made by psychiatrists based on ICD-10 criteria.

**Results:**

Among 6431 children included in the study, 309 (4.80%) were diagnosed with ADHD and 6122 served as controls. Children with ADHD showed thinner p-RNFL, m-GCL, and p-GCL than controls (*P* = 0.002; *P* = 0.03; *P* = 0.003, respectively). Among the 309 ADHD, 149 were categorized as the combined (C) type, 74 as the Inattentive (IA) type, 39 as the hyperactive/impulsive (HI) type. The ADHD-C subtype exhibited thinner p-RNFL (mean difference [MD] = -2.47, *P* = 0.03), m-GCL (MD = -1.10, *P* = 0.03), and p-GCL (MD = -0.85, *P* = 0.007) than controls. The p-RNFL, m-GCL, and p-GCL thicknesses showed a decreasing trend across ADHD subtypes, being thickest in the ADHD-HI, followed by the IA, and then the C subtype (*P* = 0.04, *P* = 0.005, and *P* = 0.04, respectively). Higher SWAN-combined scores were associated with thinner p-RNFL, m-GCL, p-GCL, and m-RNFL (*P* = 0.04; *P* = 0.002; *P* = 0.005; *P* = 0.02, respectively).

**Conclusion:**

RNFL and GCL thicknesses varied across ADHD subtypes. The combined subtypes exhibited thinner measurements than controls. An overall trend toward thinner RNFL and GCL thicknesses was observed across subtypes. RNFL and GCL thicknesses were negatively associated with severity of combined ADHD symptoms.

**Clinical trial number:**

Not applicable.

**Supplementary Information:**

The online version contains supplementary material available at https://doi.org/10.1186/s12888-026-08422-1.

## Background

Attention-Deficit/Hyperactivity Disorder (ADHD) is one of the most prevalent neurodevelopmental disorders in children, with global prevalence rates estimated at 6.7–7.8% among pediatric populations [1]. The disorder often persists into adulthood, affecting approximately 2.5% of adults [2]. Clinically, ADHD is categorized into three subtypes: predominantly inattentive (ADHD-IA), predominantly hyperactive-impulsive (ADHD-HI), and combined type (ADHD-C). The latter encompasses both inattentive and hyperactive-impulsive symptoms [3]. Although these subtypes share numerous overlapping characteristics and general treatment approaches, distinct differences exist in their management that are particularly evident in the implementation of tailored educational strategies and family support systems [4]. Early identification and differentiation of ADHD subtypes are critical for optimizing therapeutic outcomes and ensuring individualized care for affected individuals.

The diagnosis and identification of ADHD and its subtypes remain challenging, primarily due to difficulties in distinguishing symptom variability and the absence of objective diagnostic tools. Clinically, the measures currently used to assess ADHD are predominantly subjective, and there is limited understanding of the distinct pathophysiological mechanisms underlying each subtype [5, 6]. Neuroimaging studies have demonstrated that ADHD is associated with structural abnormalities in the brain [7–9]. The retina, particularly the retinal nerve fibre layer (RNFL) and ganglion cell layer (GCL), is considered as an extension of the central nervous system based on its embryonic origin from the diencephalon. It has been investigated as a potential biomarker for ADHD [10, 11]. However, most studies have focused on pooled ADHD populations without differentiating between subtypes, limiting the ability to identify subtype-specific biomarkers.

To address these gaps, we conducted a population-based, nested case-control study to investigate the associations between ADHD and its subtypes with RNFL or GCL thicknesses in children.

## Methods

### Study design and population

The present study analyzed a subset from the ongoing Hong Kong Children Eye Study, which is a population-based and territory-wide study of eye conditions in Hong Kong children [12]. Children were invited to the Chinese University of Hong Kong Eye Centre for comprehensive ocular examinations and answered a standardized questionnaires according to the unified protocol. The current study excluded children with any systemic diseases (except obesity and ADHD) and ocular diseases (except refractive errors). The study adhered to the Declaration of Helsinki, and ethical approval was obtained from the Institutional Review Board of the Chinese University of Hong Kong. All children and their parents signed an informed consent form upon participating in the study.

### OCT imaging

OCT imaging was performed by Swept-source optical coherence tomography (SS-OCT) (DRI Triton; T Topcon Inc., Tokyo, Japan) in a dark room after pupil dilation. The SS-OCT incorporates a light source at 1,050 nm wavelength and operates at a speed of 100,000 A-scans per second. Peripapillary RNFL (p-RNFL) thickness was measured by a peripapillary circle scan in a 3.4-mm diameter section around the optic nerve disc. The evaluated p-RNFL thickness parameters were average thickness, RNFL by quadrant, and 12 clock-hour sectors. Likewise, the topographical data of GCL was evaluated. The macular RNFL (m-RNFL) and macular GCL (m-GCL) thickness were measured in the following 6 sectors around the macula foveal center: nasal superior, superior, temporal superior, temporal inferior, inferior, and nasal inferior regions. Images with poor quality and signal strength index (SSI) < 50 were excluded.

### Identification of children with ADHD and assessment of ADHD symptoms

As described in our previous study, ADHD diagnoses and prescription records were retrieved from the Hong Kong Hospital Authority (HA) Electronic Medical Record (EMR) system and were established by psychiatrists based on ICD-10 criteria [13]. Participants diagnosed with ADHD were included in the ADHD group, whereas those without ADHD served as controls. Children with ADHD who had been treated with methylphenidate or amphetamine for at least one month prior to the OCT examination were defined as having a stimulant treatment history. Regarding ADHD subtype information, subtype classification is not mandatory, and documentation practices vary among psychiatrists, which has led to a lack of subtype data in the EMR system. Therefore, we used the parent-reported Strengths and Weaknesses of ADHD-Symptoms and Normal-Behaviors Questionnaire (SWAN) questionnaire to classify ADHD subtypes [14, 15]. 

The SWAN consists of 18 items rated on a 7-point scale. The average score across all items provides the ADHD-combined score, while the averages of the first nine and last nine items yield the inattentive and hyperactive/impulsive subscale scores, respectively, with higher scores indicating more severe symptoms [16]. The SWAN scale has been validated in the Hong Kong population. Cutoff scores for subtypes have been established by psychiatrists, with thresholds of 0.72 and 0.83 for IA and HI, respectively, among males, and 0.94 and 0.61 among females. Participants were classified as the ADHD-C if both criteria were met [15]. In this study, we applied these established cutoff values to classify ADHD subtypes among children with psychiatrist-diagnosed ADHD.

### Ocular examinations

Ocular biometry, including axial length (AL), was measured through noncontact partial-coherence laser interferometry (IOL Master; Carl Zeiss Meditec, Oberkochen, Germany). Refractive status was measured both before and after cycloplegia using an auto-refractor (Nidek ARK-510 A, Gamagori, Japan) for each child. An ophthalmologist made detailed examinations of the anterior segment using a slit-lamp (Haag-Streit, Koeniz, Switzerland) and the retina using a 20-D lens (Volk, Houston, TX).

### Statistical analysis

Continuous variables are reported as means with standard deviations (SDs), and categorical variables as counts with percentages. Group comparisons were performed using chi-square tests for categorical variables. For normally distributed continuous variables, Student’s t-tests were used for comparisons between two groups, and analysis of variance (ANOVA) for comparisons among more than two groups. Analyses of spherical equivalent, axial length, and RNFL and GCL thickness were performed using binocular data. Generalized estimating equations (GEE) were employed to account for inter-eye correlations within participants. GEE linear regression models were used: (1) to compare RNFL and GCL thicknesses between the ADHD subtypes and control groups, and (2) to assess associations between RNFL and GCL thicknesses and the severity of ADHD symptoms. These models were adjusted for age, sex, and AL. For comparisons among the three ADHD subtypes and controls, a GEE linear regression model was fitted, and post hoc pairwise comparisons were performed with false discovery rate (FDR) method. All statistical analyses were conducted using R software (version 4.5.2). A two-sided *P*-value of less than 0.05 was considered statistically significant.

## Results

### RNFL and GCL thicknesses in children diagnosed as ADHD

A total of 6431 children were included, comprising 309 (4.80%) children diagnosed with ADHD and 6122 children as controls, mean age of ADHD children was 7.48 ± 0.92 years, compared to 7.28 ± 0.94 years of the controls (*P* < 0.001). The proportion of males was significantly higher in the ADHD group (76.70%) than in the control group (49.71%) (*P* < 0.001) (Table 1). Among the ADHD group, 53 cases (17.15%) had a stimulant treatment history. Children with ADHD showed thinner p-RNFL (110.48 ± 13.18 vs. 112.35 ± 12.06, *P* = 0.002) than controls after adjusting for age, sex and AL. Similarly, thinner m-GCL (78.73 ± 6.15 vs. 79.14 ± 5.95, *P* = 0.03) and p-GCL (42.67 ± 4.45 vs. 43.28 ± 4.57, *P* = 0.003) were observed in ADHD (Table 1).

**Table 1:** Basic information and RNFL and GCL thicknesses in ADHD and controls

	ADHD (*n* = 309)	Control (*n* = 6122)	*P* value
Age, mean (SD), year	7.48 (0.92)	7.28 (0.94)	< 0.001
Male, No. (%)	237 (76.70)	3042 (49.71)	< 0.001
Axial length^a^, mean (SD), mm	23.16 (0.98)	23.02 (0.93)	0.03
Spherical equivalent^a^, mean (SD), diopter	0.01 (1.75)	0.22 (1.48)	0.22
p-RNFL^b^, mean (SD), um	110.48 (13.18)	112.35 (12.06)	0.002
m-GCL^b^, mean (SD), um	78.73 (6.15)	79.14 (5.95)	0.03
p-GCL^b^, mean (SD), um	42.67 (4.45)	43.28 (4.57)	0.003
m-RNFL^b^, mean (SD), um	31.56 (2.93)	31.77 (2.63)	0.06

^**a**^ Using binocular data and correcting for binocular correlation with GEE model, adjusted for age and sex. ^**b**^ Using binocular data and correcting for binocular correlation with GEE model, adjusted for age, sex and axial length

### RNFL and GCL thicknesses in ADHD subtypes

Among the 309 children with ADHD, 149 were categorized as the ADHD-C type, 74 as the IA type, 39 as the HI type. The remaining 47 children could not be categorized, as their SWAN subscale scores fell below the predefined cutoff thresholds and were therefore excluded from the subtype analysis. Comparisons between these 47 unclassifiable ADHD and children with classified ADHD subtypes showed no differences in OCT parameters (Supplementary Table 1). Overall comparisons revealed significant differences in p-RNFL, m-GCL, and p-GCL thicknesses among the three ADHD subtypes and controls (*P* = 0.02, *P* = 0.009, *P* = 0.01, respectively) (Table 2).

**Table 2:** Basic information and RNFL and GCL thicknesses among ADHD subtypes and controls

	(1) ADHD-C (*n* = 149)	(2) ADHD-IA (*n* = 74)	(3) ADHD-HI (*n* = 39)	(4) Control (*n* = 6122)	*P* value^c^
Age, mean (SD), year	7.40 (0.86)	7.64 (0.93)	7.29 (1.00)	7.28 (0.94)	0.005
Male, No. (%)	118 (79.19)	59 (79.73)	21 (53.85)	3043 (49.71)	< 0.001
Axial length^a^, mean (SD), mm	23.15 (0.97)	23.05 (1.10)	23.06 (0.91)	23.02 (0.93)	0.02
Spherical equivalent^a^, mean (SD), diopter	0.01 (1.68)	0.18 (1.94)	0.18 (1.39)	0.22 (1.48)	0.52
P-RNFL^b^, mean (SD), um	109.66 (15.02)	110.98 (11.09)	112.75 (10.34)	112.35 (12.06)	0.02 (1) vs. (4) P^d^ = 0.03
m-GCL^b^, mean (SD), um	78.20 (7.21)	78.97 (4.21)	80.10 (5.06)	79.14 (5.95)	0.009 (1) vs. (4) P^d^ = 0.03 (1) vs. (3) P^d^ = 0.02 (2) vs. (3) P^d^ = 0.04
p-GCL^b^, mean (SD), um	42.36 (4.54)	42.99 (4.33)	43.39 (4.76)	43.28 (4.57)	0.01 (1) vs. (4) P^d^ = 0.008
m-RNFL^b^, mean (SD), um	31.47 (3.64)	31.47 (1.93)	32.05 (2.03)	31.77 (2.63)	0.11

ADHD-C: combined ADHD subtype, ADHD-IA: inattentive ADHD subtype, ADHD-HI: hyperactive/impulsive ADHD subtype. ^**a**^ Using binocular data and correcting for binocular correlation with GEE model, adjusted for age and sex. ^**b**^ Using binocular data and correcting for binocular correlation with GEE model, adjusted for age, sex and axial length. ^c^ P values refer to overall comparisons across the four groups. ^d^ Post hoc pairwise comparisons with significance are presented after FDR adjustment

**Fig. 1:**
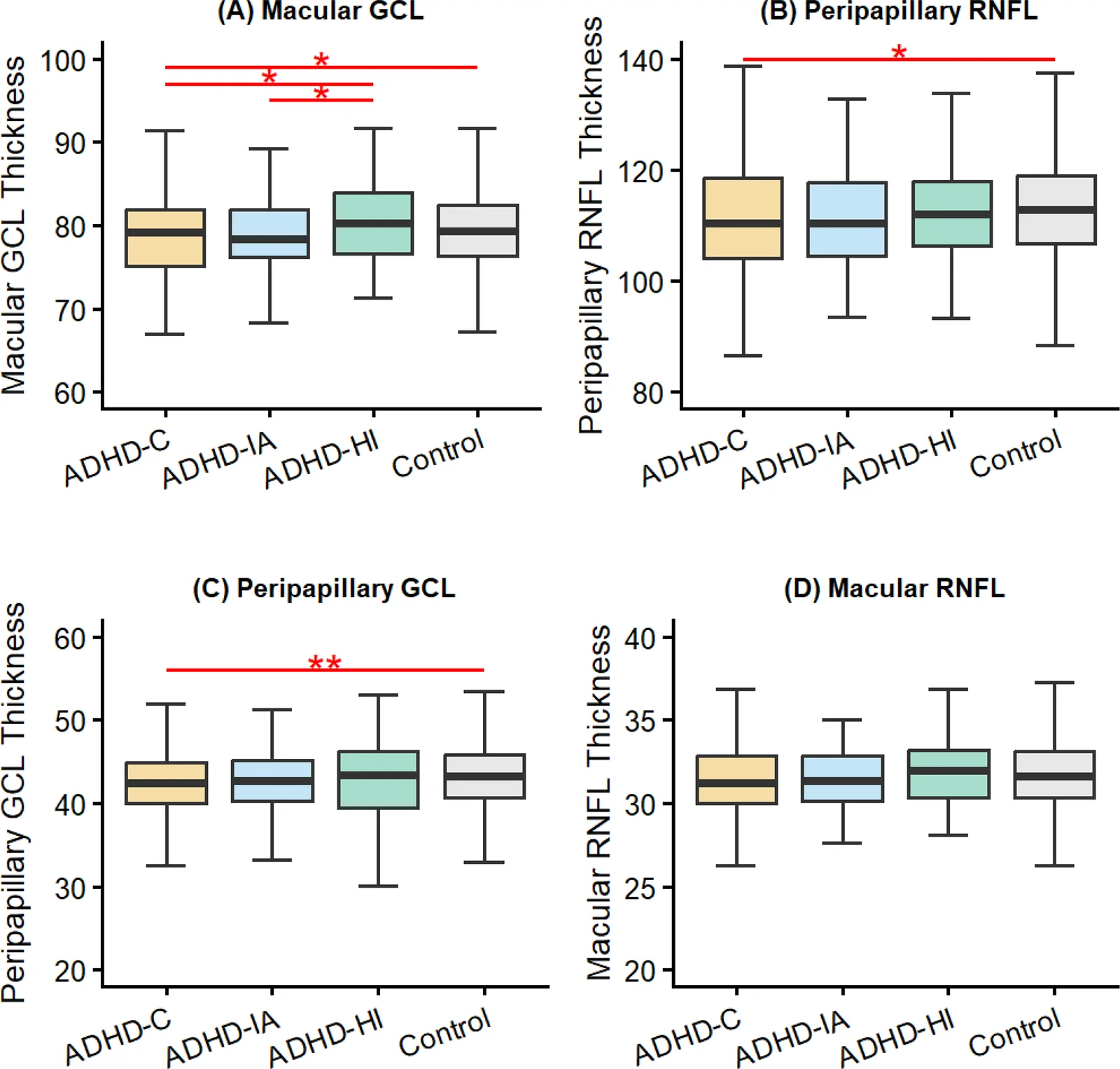
Post-hoc Comparisons of RNFL and GCL Thicknesses between ADHD Subtypes and Controls Footnote below the figure: Asterisks indicate statistically significant differences between groups based on post hoc pairwise comparisons with FDR correction (*P < 0.05, **P < 0.01)

In post-hoc pairwise comparisons with FDR correction, the ADHD-C subtype exhibited significantly thinner p-RNFL (mean difference [MD] = -2.47, *P* = 0.03), m-GCL (MD = -1.10, *P* = 0.03), and p-GCL (MD = -0.85, *P* = 0.007) than controls. The ADHD-HI subtype showed thicker m-GCL than the ADHD-C (MD = 2.05, *P* = 0.02) and the ADHD-IA (MD = 1.47, *P* = 0.04) (Table 2; Fig. 1). The p-RNFL, m-GCL, and p-GCL thicknesses showed a decreasing trend across ADHD subtypes, being thickest in ADHD-HI, followed by the IA, and then the C subtype (*P* = 0.04, *P* = 0.005, and *P* = 0.04, respectively). The RNFL and GCL thicknesses in the superior, inferior, nasal, and temporal regions of the peripapillary and macular areas among ADHD subtypes and controls are presented in Table 3.

**Table 3:** Subregions RNFL and GCL thicknesses among ADHD subtypes and controls

	(1) ADHD-C (*n* = 149)	(2) ADHD-IA (*n* = 74)	(3) ADHD-HI (*n* = 39)	(4) Control (*n* = 6122)	*P* value^b^
**p-RNFL**^**a**^, **mean (SD), um**					
Superior Region	141.28 (22.62)	143.83 (20.82)	144.36 (16.06)	144.03 (20.14)	0.22
Inferior Region	140.16 (25.15)	140.49 (16.28)	143.43 (16.05)	143.55 (20.30)	0.02
Nasal Region	72.99 (15.64)	74.46 (12.60)	73.03 (14.93)	75.08 (14.44)	0.04
Temporal Region	84.20 (15.05)	85.15 (11.19)	90.05 (15.52)	86.64 (13.68)	0.02 (1) vs. (3) P^c^ = 0.03
**m-GCL**^**a**^, **mean (SD)**,** um**					
Superior Region	78.15 (8.14)	79.20 (4.74)	80.57 (4.88)	79.64 (6.48)	< 0.001 (1) vs. (4) P^c^ = 0.003 (1) vs. (3) P^c^ = 0.003 (2) vs. (3) P^c^ = 0.02
Inferior Region	76.50 (7.20)	77.07 (4.43)	78.11 (4.95)	77.36 (6.18)	0.03
Nasal Region	80.96 (9.06)	81.53 (5.50)	82.51 (6.50)	81.51 (8.03)	0.27
Temporal Region	77.19 (9.18)	78.09 (5.86)	79.20 (5.45)	78.03 (7.44)	0.03 (1) vs. (3) P^c^ = 0.03
**p-GCL**^**a**^, **mean (SD)**,** um**					
Superior Region	39.25 (6.27)	40.06 (5.37)	40.85 (6.05)	40.38 (6.07)	0.01 (1) vs. (4) P^c^ = 0.01
Inferior Region	38.02 (5.58)	39.20 (7.19)	39.48 (5.61)	39.19 (5.62)	0.01 (1) vs. (4) P^c^ = 0.006
Nasal Region	41.81 (5.42)	42.27 (3.74)	42.54 (5.77)	42.57 (4.92)	0.17
Temporal Region	50.37 (6.68)	50.48 (5.21)	50.47 (7.39)	50.99 (7.41)	0.28
**m-RNFL**^**a**^, **mean (SD)**,** um**					
Superior Region	32.77 (4.91)	33.14 (2.31)	33.79 (2.66)	33.45 (3.30)	0.11
Inferior Region	34.16 (3.77)	34.10 (2.81)	34.80 (2.70)	34.50 (3.65)	0.20
Nasal Region	36.13 (5.86)	36.00 (3.02)	36.88 (3.04)	36.34 (4.05)	0.20
Temporal Region	22.71 (3.06)	22.64 (1.36)	22.71 (0.92)	22.79 (1.96)	0.44

ADHD-C: combined ADHD subtype, ADHD-IA: inattentive ADHD subtype, ADHD-HI: hyperactive/impulsive ADHD subtype. ^**a**^ Using binocular data and correcting for binocular correlation with GEE model, adjusted for age, sex and axial length. ^b^ P values refer to overall comparisons across the four groups. ^c^ Post hoc pairwise comparisons with significance are presented after FDR adjustment

### Association between severity of ADHD symptoms and RNFL and GCL thicknesses

Among the overall population, higher SWAN-combined scores was associated with thinner p-RNFL (β = -0.21 μm/score, *P* = 0.04), m-GCL (β = -0.15 μm/score, *P* = 0.002), p-GCL (β = -0.12 μm/score, *P* = 0.005), and m-RNFL thicknesses (β = -0.05 μm/score, *P* = 0.02) (Table 4). In contrast, SWAN-inattentive scores showed borderline but non-significant associations with thinner m-GCL (β = -0.13 μm/score, *P* = 0.07), p-GCL (β = -0.11 μm/score, *P* = 0.09), and m-RNFL (β = -0.06 μm/score, *P* = 0.06). No significant associations were observed between SWAN-hyperactivity/impulsivity scores and RNFL or GCL thickness (Table 4).

**Table 4:** Association between severity of ADHD symptoms and RNFL and GCL thicknesses

	*p*-RNFL		m-GCL		*p*-GCL		m-RNFL	
	β (95% CI)	*P* value^a^	β (95% CI)	*P* value^a^	β (95% CI)	*P* value^a^	β (95% CI)	*P* value^a^
SWAN combined Score^b, c^	-0.21 (-0.41 to -0.01)	0.04	-0.15 (-0.24 to -0.05)	0.002	-0.12 (-0.20 to -0.03)	0.005	-0.05 (-0.09 to -0.01)	0.02
SWAN Inattentive Score^b, d^	-0.24 (-0.54 to 0.05)	0.11	-0.13 (-0.27 to 0.01)	0.07	-0.11 (-0.23 to 0.02)	0.09	-0.06 (-0.12 to 0.002)	0.06
SWAN Hyperactivive/Impulsive Score^b, d^	0.01 (-0.25 to 0.27)	0.93	-0.02 (-0.15 to 0.10)	0.73	-0.02 (-0.13 to 0.10)	0.79	0.004 (-0.05 to 0.06)	0.89

^**a**^ Analyses based on full-population binocular data, using GEE model to correct for interocular correlation. ^**b**^ The SWAN combined score was calculated as the mean of all 18 items. The inattention score was calculated as the mean of the first nine items, and the hyperactivity/impulsivity score was calculated as the mean of the last nine items. ^C^ Combined scores were included as the independent variable, with RNFL and GCL thickness as dependent variables, adjusting for age, sex, and axial length. ^d^ Inattentive and hyperactive/impulsive scores were included as the independent variable in one model, with RNFL and GCL thickness as dependent variables, adjusting for age, sex, and axial length

## Discussion

In this study, we found that RNFL and GCL thicknesses were reduced in children with ADHD. Notably, we observed variations in these changes across different ADHD subtypes. ADHD-C exhibited thinner RNFL and GCL than controls. RNFL and GCL thicknesses appeared to decrease across subtypes; however, given the unbalanced group sizes and limited effective size, these findings should be interpreted with caution. Confirmatory studies with larger ADHD-HI samples are needed before any biological interpretation can be made. Furthermore, RNFL and GCL thicknesses were negatively correlated with the severity of ADHD symptoms. Taken together, our data reveal that RNFL and GCL thicknesses are significantly associated with ADHD, with variations observed across subtypes and symptom severity. Differences in these retinal layers between ADHD subtypes may help elucidate their underlying pathophysiology. These findings provide preliminary evidence supporting further investigation of retinal indices as potential biomarkers of ADHD.

This study used the retina as a platform to study disorders in the central nerve system (CNS) because it is an accessible extension of the brain in terms of embryology, anatomy, and physiology [10]. Emerging evidence suggests an association between ADHD and neuropathology, similar to seizure disorders [17–19]. The RNFL contains ganglion cells with unmyelinated axons, which are considered to be an extension of the cerebral gray matter. Our findings of RNFL and GCL thinning in ADHD children align with previous MRI findings of reduced cerebral gray matter in individuals with ADHD [20–23]. Hergüner et al. also found that reduced RNFL thickness in children with ADHD [24]. Children and adolescents with ADHD and Oppositional Defiant Disorder (ODD) accompanying ADHD had reportedly thinner GCL [25]. 

Not much information has been reported on the differences of retinal morphometric imaging between subtypes of ADHD. Clinically, ADHD-C is the most common subtype, whereas ADHD-HI is relatively rare. The symptoms of these subtypes differ significantly. ADHD-IA is characterized by symptoms of distractibility, forgetfulness, and difficulty sustaining attention, which are markedly different from the hyperactivity and impulsivity observed in ADHD-HI. Moreover, these subtypes may possess distinct neuroanatomical and functional profiles [26–28]. A case-control study reported that the severe-combined with emotional dysregulation ADHD exhibited widespread medial prefrontal cortex-pallidum alterations, whereas the predominantly hyperactive/impulsive ADHD demonstrated anterior cingulate cortex-pallidum circuit alterations, and the predominantly inattentive ADHD showed superior frontal gyrus alterations [29]. Semrud-Clikeman et al. found that ADHD combined children had bilaterally smaller volumes of the caudate and anterior cingulate cortex than neurotypical peers [30]. Similarly, ShuHua et al. reported decreased subcortical volume in patients with ADHD combined type compared with neurotypical peers [31]. These results are consistent with our finding that children with ADHD-C had thinner RNFL and GCL thicknesses than controls. We speculate that the differences in RNFL and GCL thicknesses among ADHD subtypes reflect variations in underlying neurodevelopmental processes. Further research and validation are warranted to confirm these findings and elucidate the underlying mechanisms.

We found that RNFL and GCL thicknesses were negatively associated with the severity of ADHD symptoms. In a cohort study of 2235 healthy cases, there was association between thinner RNFL and Ganglion Cell Complex (GCC) with lower gray matter density in the primary visual cortex [32]. Retinal thinning observed in ADHD may reflect alterations in neuronal integrity along the visual pathway. While anterograde degeneration has been proposed as one possible mechanism [33], alternative explanations include broader neurodevelopmental alterations associated with ADHD, as well as retrograde transsynaptic effects from central visual and attentional networks [34]. Interestingly, when analyzed separately by symptom domains, the observed effect appeared to be primarily driven by inattentive symptoms, showing a borderline significance, whereas no association was found for hyperactive/impulsive symptoms. Study have reported increased grey matter volume (GMV) involving the frontal regions and cerebellum with increasing inattentiveness severity [35]. In contrast, greater overall ADHD severity has been associated with grey matter atrophy in regions including the cerebellum and hippocampus. These findings suggest that brain structural alterations in ADHD may be heterogeneous and dependent on symptom domain and severity, which may partly explain the observed retinal changes. Further studies are needed to elucidate the underlying mechanisms.

Strengths of the present study are the large sample size and population-based design. The diagnosis of ADHD was confirmed by psychiatrists according to ICD-10 criteria [36]. This sample size enables the generalizability of our findings among different subtypes of ADHD. However, there are several limitations in current study. First, ADHD subtypes were classified using the SWAN questionnaire. Although the SWAN is a well-validated instrument, some degree of misclassification is possible because subtype assignment was based on parent-reported symptoms rather than clinician-determined diagnoses. Second, a proportion of children with ADHD had a history of stimulant treatment. Although stimulant use did not influence the association between ADHD subtypes and OCT indices in the primary analysis and was therefore not included in the final analysis, its potential impact on OCT indices cannot be completely ruled out. Future longitudinal studies are warranted to further clarify the relationship between stimulant treatment and retinal structural changes. Third, while this study examined retinal thickness across ADHD subtypes, reverse causation and shared underlying neurodevelopmental factors cannot be ruled out. Longitudinal studies and mechanistic investigations are needed to clarify these relationships. Lastly, the observed group differences and associations, although statistically significant, were relatively modest in magnitude. Therefore, the interpretation of the clinical meaning needs to be cautious.

In conclusion, RNFL and GCL thickness varied across ADHD subtypes. The combined subtypes exhibited significantly thinner measurements than controls. An overall trend toward thinner RNFL and GCL thicknesses was observed across subtypes. Furthermore, RNFL and GCL thickness were negatively associated with severity of combined ADHD symptoms. These findings, which may help elucidate the pathophysiology of ADHD subtypes, provide preliminary evidence supporting further investigation of retinal indices as potential biomarkers of ADHD.

## Supplementary Information

Below is the link to the electronic supplementary material.

Supplementary Material 1

## Data Availability

The datasets used and/or analysed during the current study are available from the corresponding author on reasonable request.

## Abbreviations

ADHD: Attention Deficit Hyperactivity Disorder
RNFL: Retinal Nerve Fibre Layer
GCL: Ganglion Cell Layer
SS-OCT: Swept-source optical coherence tomography
HA: Hospital Authority
EMR: Electronic Medical Records
SWAN: Strengths and Weaknesses of ADHD-symptoms and Normal-behaviors Questionnaire
AL: Axial Length
SD: Standard Deviations
ANOVA: Analysis of Variance
GEE: Generalized Estimating Equations
