# Supplementary material for: Retinal nerve fibre layer, and ganglion cell layer thicknesses in children with ADHD: associations with subtypes and symptom severity
Source: BMC Psychiatry. 2026 Aug 7;26:866. doi: 10.1186/s12888-026-08422-1 (PMC13733042; doi:10.1186/s12888-026-08422-1)
Supplement: Supplementary file 1 [file 12888_2026_8422_MOESM1_ESM.docx]

**Table S1. Basic Information and RNFL and GCL Thicknesses in ADHD**

|  | Unclassifiable ADHD (n=47) | Classifiable ADHD (n=262) | P value |
| --- | --- | --- | --- |
| Age, mean (SD), year | 7.65 (0.99) | 7.45 (0.91) | 0.16 |
| Male, No. (%) | 39 (82.98%) | 198 (75.57%) | 0.36 |
| Axial length^a^, mean (SD), mm | 23.41 (0.87) | 23.11 (1.00) | 0.02 |
| Spherical equivalent^a^, mean (SD), Diopter | -0.36 (1.88) | 0.08 (1.72) | 0.06 |
| p-RNFL^b^, mean (SD), um | 110.41 (12.03) | 110.50 (13.40) | 0.75 |
| m-GCL^b^, mean (SD), um | 78.85 (5.83) | 78.71 (6.22) | 0.61 |
| p-GCL^b^, mean (SD), um | 42.54 (4.09) | 42.69 (4.52) | 0.67 |
| m-RNFL^b^, mean (SD), um | 31.60 (2.35) | 31.56 (3.03) | 0.71 |

**^a^** Using binocular data and correcting for binocular correlation with GEE model, adjusted for age and sex. **^b^** Using binocular data and correcting for binocular correlation with GEE model, adjusted for age, sex and axial length.
